# RETRACTION: Changes in Immunological Parameters in Patients Treated Using Direct and Indirect Restorations of the Hard Tissues of the Anterior Teeth in Combination With an Antioxidant

**DOI:** 10.1155/term/9815150

**Published:** 2025-03-15

**Authors:** Journal of Tissue Engineering and Regenerative Medicine

RETRACTION: I. R. Kumhyr, V. P. Levko, and Z. R. Ozhogan, “Changes in Immunological Parameters in Patients Treated Using Direct and Indirect Restorations of the Hard Tissues of the Anterior Teeth in Combination With an Antioxidant,” *Journal of Tissue Engineering and Regenerative Medicine*, no. 14 (2020): 1001–1005, https://doi.org/10.1002/term.3054.

The above article, published online on 29 April 2020 in Wiley Online Library (https://wileyonlinelibrary.com), has been retracted by agreement between the Chief Editor, Catherine K. Kuo, and John Wiley & Sons Ltd. UK. The retraction has been agreed following concerns raised by a third party regarding the peer review process. Further investigation by the publisher has found manipulation of the peer review process. The authors did not respond to requests for an explanation. As a result, the conclusions reported in the article are not considered reliable.

